# Defects in ErbB-Dependent Establishment of Adult Melanocyte Stem Cells Reveal Independent Origins for Embryonic and Regeneration Melanocytes

**DOI:** 10.1371/journal.pgen.1000544

**Published:** 2009-07-03

**Authors:** Keith A. Hultman, Erine H. Budi, Daniel C. Teasley, Andrew Y. Gottlieb, David M. Parichy, Stephen L. Johnson

**Affiliations:** 1 Department of Genetics, Washington University School of Medicine, St. Louis, Missouri, United States of America; 2 Department of Biology, Institute for Stem Cell and Regenerative Medicine, University of Washington, Seattle, Washington, United States of America; University of Bath, United Kingdom

## Abstract

Adult stem cells are responsible for maintaining and repairing tissues during the life of an organism. Tissue repair in humans, however, is limited compared to the regenerative capabilities of other vertebrates, such as the zebrafish (*Danio rerio*). An understanding of stem cell mechanisms, such as how they are established, their self-renewal properties, and their recruitment to produce new cells is therefore important for the application of regenerative medicine. We use larval melanocyte regeneration following treatment with the melanocytotoxic drug MoTP to investigate these mechanisms in Melanocyte Stem Cell (MSC) regulation. In this paper, we show that the receptor tyrosine kinase, erbb3b, is required for establishing the adult MSC responsible for regenerating the larval melanocyte population. Both the erbb3b mutant and wild-type fish treated with the ErbB inhibitor, AG1478, develop normal embryonic melanocytes but fail to regenerate melanocytes after MoTP-induced melanocyte ablation. By administering AG1478 at different time points, we show that ErbB signaling is only required for regeneration prior to MoTP treatment and before 48 hours of development, consistent with a role in establishing MSCs. We then show that overexpression of kitla, the Kit ligand, in transgenic larvae leads to recruitment of MSCs, resulting in overproliferation of melanocytes. Furthermore, kitla overexpression can rescue AG1478-blocked regeneration, suggesting that ErbB signaling is required to promote the progression and specification of the MSC from a pre–MSC state. This study provides evidence that ErbB signaling is required for the establishment of adult MSCs during embryonic development. That this requirement is not shared with the embryonic melanocytes suggests that embryonic melanocytes develop directly, without proceeding through the ErbB-dependent MSC. Moreover, the shared requirement of larval melanocyte regeneration and metamorphic melanocytes that develops at the larval-to-adult transition suggests that these post-embryonic melanocytes develop from the same adult MSC population. Lastly, that *kitla* overexpression can recruit the MSC to develop excess melanocytes raises the possibility that Kit signaling may be involved in MSC recruitment during regeneration.

## Author Summary

The promise of regenerative medicine lies in the ability to find or create stem cells that can be manipulated to replace damaged tissues and organs. Such ability requires an understanding of how adult stem cells are established and then later recruited to regenerate different tissues. Here, we study the zebrafish's ability to regenerate melanocytes, a pigment cell shared with humans, to understand these mechanisms. We show that the *erbb3b* gene is required to establish melanocyte stem cells in the embryo that are responsible for regenerating melanocytes after melanocytes are ablated in the larval zebrafish. Because this adult stem cell is not required for the development of embryonic melanocytes, we conclude that adult melanocyte stem cells develop in parallel to the embryonic tissues that they regulate. We also show that overexpressing *kit ligand a* will result in over-recruiting these adult stem cells to produce excess melanocytes. Further exploration into the mechanisms by which the zebrafish melanocyte stem cells are maintained and recruited will inform how adult stem cells might be manipulated for medical applications.

## Introduction

As with other teleosts, the zebrafish has a remarkable ability to regenerate a wide range of tissues and organs including fins [1], heart [2], and retina [3]–[5]. Epimorphic regeneration, involving the cell division and differentiation of undifferentiated post-embryonic precursors, or adult stem cells, has been proposed for each of these cases. Epimorphic regeneration requires several distinct events for the function of the adult stem cell: establishment, self-renewal, recruitment, and differentiation. In order to understand regeneration and its practical application in medicine, it is important to understand the molecular processes underlying each of these events [6]. Very little is known about what developmental mechanisms are required for establishing adult stem cells. Furthermore, the relationship between adult stem cells and the primary cells or tissue they regulate is not yet clearly understood. It is unknown, for instance, whether adult stem cells contribute to embryonic growth and then remain quiescent until later recruitment, or if they are established by developmental mechanisms that are distinct from embryonic precursors.

The zebrafish embryonic pigment pattern is an excellent genetic model for investigating developmental processes [7]. Of the neural crest derived pigmented cells, the melanocyte is of particular interest as it is common to both zebrafish and human, and several genetic pathways are conserved between the two [8]–[10]. The embryonic melanocyte lineage arises from the neural crest beginning at 14 hours post fertilization (hpf). The melanocyte precursors, or melanoblasts, are not yet pigmented and migrate to the periphery where they differentiate and begin to melanize by 24 hpf. Most of the embryonic population of melanocytes is established by 3 dpf comprised of approximately 460 melanocytes [10]. Except for approximately 20 additional melanocytes that develop from 3–8 dpf at the horizontal myoseptum [11], melanocyte number remains static, with little to no turnover, until the adult pigment pattern is formed during metamorphosis at ∼14 dpf [12].

We have developed a model for cell-specific regeneration of melanocytes during the period of stasis between 3 and 14 dpf [13]. The melanocytotoxic chemical 4-(4-morpholinobutylthio)phenol (MoTP) is converted into a cytotoxin by the melanin synthesis enzyme tyrosinase. Accordingly, MoTP specifically ablates cells that express tyrosinase at high levels, which in the embryo are limited to melanoblasts and newly formed melanocytes. Typically MoTP is applied for 2 days to completely ablate the embryonic melanocyte lineage [13]. BrdU incorporation studies in regenerated melanocytes indicate that stem cells begin to divide to replace dying melanocytes within 24 hours of MoTP exposure. Regeneration is complete within 3–4 days after MoTP washout, eventually replacing melanocytes in all parts of the larvae except the ventral-most yolk stripe. This procedure has allowed us to identify roles for previously identified genes, such as the receptor tyrosine kinase *kit* [13] in the development of melanocytes from the melanocyte stem cell (MSC), as well as genes with regeneration-specific functions that act at early and late stages of melanocyte regeneration [14]. *kit* acts cell autonomously in the melanocyte lineage [10], responding to its ligand (*kitla* in zebrafish) expressed in the skin or in cells adjacent to the notochord [15] to promote migration [10],[15],[16], survival [10],[15],[16], differentiation [17] and possibly cell division at different stages of the melanocyte lineage. In mouse, in addition to well described roles in the melanoblast and melanocyte, Kit is also expressed in the MSC [18], although the role of its expression in the MSC has not been explored. In zebrafish, roles for *kit* signaling have been demonstrated for both larval [19] and adult [20] melanocyte regeneration. It is not yet known whether this requirement for *kit* is in the MSC itself, to promote recruitment of these quiescent stem cells, or for subsequent differentiation of regeneration melanoblasts, or both.

Genes identified by mutant phenotypes expressed during metamorphosis of the embryonic or early larval pigment pattern to the adult pigment pattern may also be involved in processes of adult stem cell regulation [21]–[23]. Metamorphosis mutants *puma* and *picasso* each show deficits in forming new melanocytes at the onset of metamorphosis. The positional cloning of *picasso* revealed that it was caused by a mutation in *erbb3b*, an epidermal growth factor receptor (EGFR)-like tyrosine kinase. Furthermore, drug inhibitors of the ErbB family of receptors, such as AG1478, administered to WT larvae as early as 14–22 hpf phenocopied the *picasso* metamorphic defect. This finding strongly suggested a role for ErbB signaling during early embryonic development in establishing the stem cells involved in adult melanocyte development. An attractive possibility is that ErbB-dependent stem cells responsible for metamorphosis are also responsible for surveilling and regulating the larval melanocyte population.

In this study, we explore the role of ErbB signaling in establishing MSC populations that are drawn on for larval melanocyte regeneration. We show that both the *picasso* mutation and the ErbB inhibitor, AG1478, abolish melanocyte regeneration following embryonic melanocyte ablation by MoTP. Our results indicate a requirement of ErbB activity concurrent with embryonic melanocyte development and prior to melanocyte ablation by MoTP, suggesting that ErbB signaling is required during the establishment of larval MSCs. That both larval regeneration and metamorphosis require early ErbB signaling raises the possibility that the same stem cell population is involved in both larval regeneration and metamorphosis.

Furthermore, the finding that *picasso* or ErbB inhibitor treated embryos have normal embryonic pigment pattern but lack functional stem cells involved in regeneration or metamorphosis indicates there are two types of melanocytes: secondary larval regeneration melanocytes that develop from an ErbB-dependent stem cell, and primary ontogenetic, or embryonic, melanocytes that develop independent of ErbB function.

We also examined how AG1478 affects the establishment of MSCs. We took advantage of a transgenic line with constitutive overexpression of the *kit ligand a*, *Tg(cmv:kitla)*, which causes an increase in the number of ontogenetic melanocytes. We investigated which melanocyte lineage is responsible for increased melanocytes by examining the effects of drugs that specifically affect the stem-cell derived melanocytes. Thus, we show that the drug ICI-118,551, which specifically blocks regeneration melanocyte development at the *dct+* melanoblast stage but has no effect on the development of embryonic or ontogenetic melanocytes, ablates the *kitla*-mediated melanocyte increase in *Tg(cmv:kitla)* larvae. Moreover, AG1478, that blocks the establishment of functional MSCs, when given to *Tg(cmv:kitla)* larvae results in an additional increase in ectopic melanocytes. This additional increase is also blocked by the regeneration specific drug, ICI-118,551. That both regeneration-specific drugs have an effect on the number of ectopic melanocytes in *Tg(cmv:kitla)* larvae is evidence that overexpression of *kitla* is acting through the MSC lineage to produce excess melanocytes. That AG1478 does not reduce the number of excess melanocytes in *Tg(cmv:kitla)* larvea suggests that AG1478 arrests the stem cell lineage in a pre-stem cell stage (pre-MSC) and that these pre-MSC cells can be activated by *kitla* overexpression.

## Results

### ErbB Signaling Is Required for Larval Melanocyte Regeneration, But Not Ontogeny

**Figure 1:**
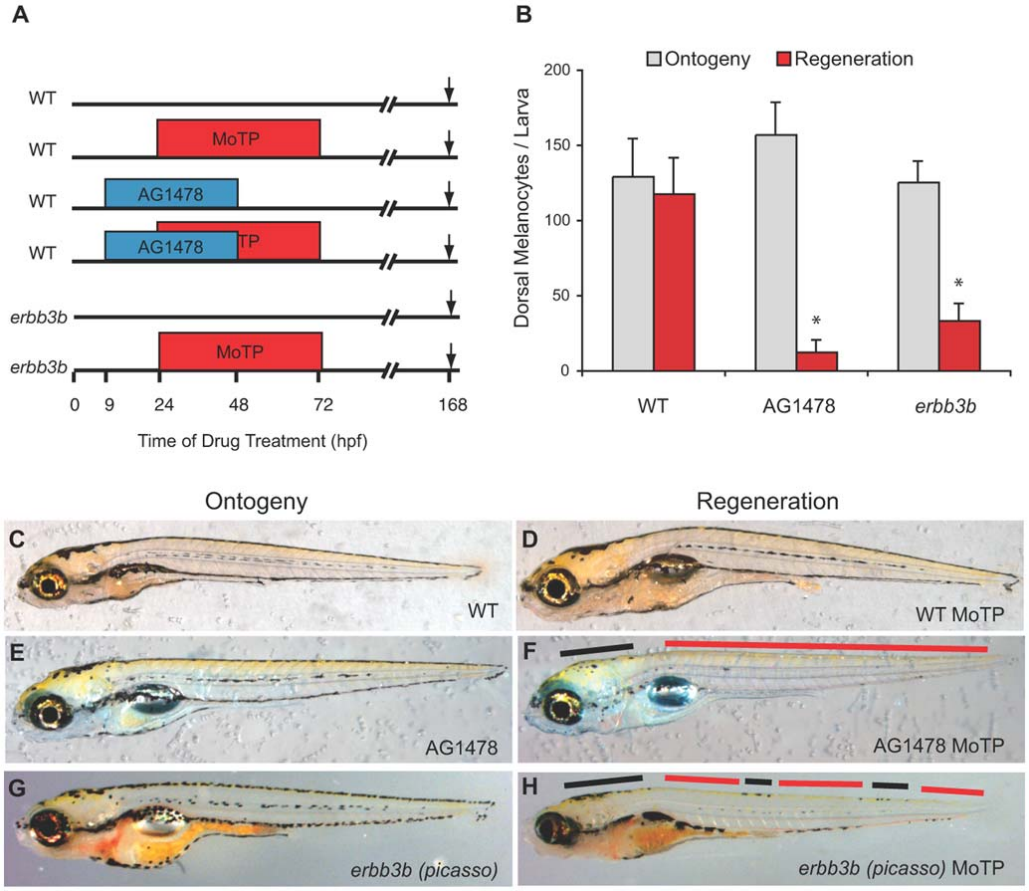
ErbB signaling is required for larval melanocyte regeneration. (A) Cartoon of drug treatment timeline for assaying melanocyte ontogeny and regeneration. Arrow indicates when embryos were collected for photos and melanocyte counts, in this case at 168 hpf. (B) Quantitation of average dorsal melanocytes from somites 1–26 for each treatment in (A) for melanocyte ontogeny (gray) and melanocyte regeneration (red). Error bars represent standard deviation, * represents P<0.05 (Student t-test, N = 10). Photos of representative larvae at 168 hpf for ontogeny (C, E, G) and regeneration (D, F, H). WT larvae regenerate nearly completely (compare D to C). WT treated with AG1478 (E, F) and *erbb3b* mutants (G, H) largely fail to regenerate but have normal ontogenetic number of melanocytes. A few regeneration melanocytes are observed in the head and sporadically in parts of the trunk (black bars in F and H) but are mostly absent throughout the trunk (red bars in F and H).

We were interested in testing whether ErbB signaling is required for larval melanocyte regeneration and ontogeny. Thus, we treated fish with the ErbB inhibitors AG1478 and PD158780 [24], or DMSO, from 9–48 hpf, during neural crest and melanocyte development. To assay for melanocyte regeneration, the fish were also incubated with the melanocyte-specific cytotoxic drug, MoTP, from 24–72 hpf (Figure 1A). Both ErbB inhibitors resulted in a deficit in the number of regenerated melanocytes but not ontogenetic melanocytes. Untreated larvae develop approximately 129.1±25.1 (mean±standard deviation) dorsal stripe melanocytes by 7 dpf (Figure 1C), and following MoTP melanocyte ablation, regenerate 91% (117.5±24.0 melanocytes) of their ontogenetic number (Figure 1D). Similarly, larvae treated with AG1478 (Figure 1E) have normal numbers (157.0±21.2) of ontogenetic melanocytes. However, when larvae are treated with both MoTP and AG1478 (Figure 1F), they regenerate less than 8% (12.4±7.8 melanocytes) of their original ontogenetic number, and significantly less than the number that regenerate following MoTP treatment alone (P<0.0001). Larva treated with PD158780 and MoTP showed a similar regeneration defect and were not significantly different from AG1478 (P = 0.5, not shown) and all subsequent ErbB inhibitor experiments were conducted with AG1478.

To test whether the gene *erbb3b* was specifically required for regeneration, we treated *erbb3b* (*picasso*) mutants with DMSO or MoTP from 24–72 hpf and assayed for melanocyte ontogeny and regeneration at 7 dpf (Figure 1A). As was previously reported [23], *erbb3b* mutants have no deficit in embryonic melanocyte ontogeny (Figure 1G). *erbb3b* animals have 125.4±13.7 dorsal and lateral melanocytes in the trunk, which is not significantly different from wild type (P = 0.6). Following melanocyte ablation with MoTP, *erbb3b* larvae also showed a defect in regeneration (Figure 1H), regenerating approximately 33.2±11.3 melanocytes, significantly less than wild-type regeneration (P<0.0001).

In both AG1478-treated larvae and *erbb3b* mutants some regeneration was observed on the head and sporadically in parts of the trunk (Figure 1F and 1H, black bars). The largest deficit in regeneration was observed in the trunk in dorsal stripe (red bars) and lateral stripe melanocytes. Curiously, the regeneration deficit was more severe in inhibitor treated larvae than in *erbb3b* mutant larvae. One possible explanation for this small increase in residual number of regeneration melanocytes in the *erbb3b* mutants (33.2±11.3 melanocytes) compared to those that regenerate in WT larvae (12.4±7.8 melanocytes) is that partial suppressors of the regeneration defect have accumulated in the mutant stocks, that act equally on the mutant and the pharmacological inhibitor. Whatever is the cause of these increased residual melanocytes, the finding that the deficit in regeneration is not increased by combining the mutant and the pharmacological inhibitor (42±18.5 melanocytes, P = 0.2) is consistent both with reports that AG1478 is highly specific to the ErbB family of receptor tyrosine kinases [25] and with several reports that AG1478 blocks *erbb2*- and *erbb3b*-dependent signaling in zebrafish [23],[24],[26]. That the inhibitor-treated mutants have no additional deficit in regeneration melanocytes than either single perturbation argues that any possible additional actions of the drug on other ErbB class receptors or other kinases have no strong effects on the establishment of the melanocyte stem cell.

### ErbB Is Required for Larval Melanocyte Stem Cell (MSC) Establishment, But Not in Recruitment or Differentiation

**Figure 2:**
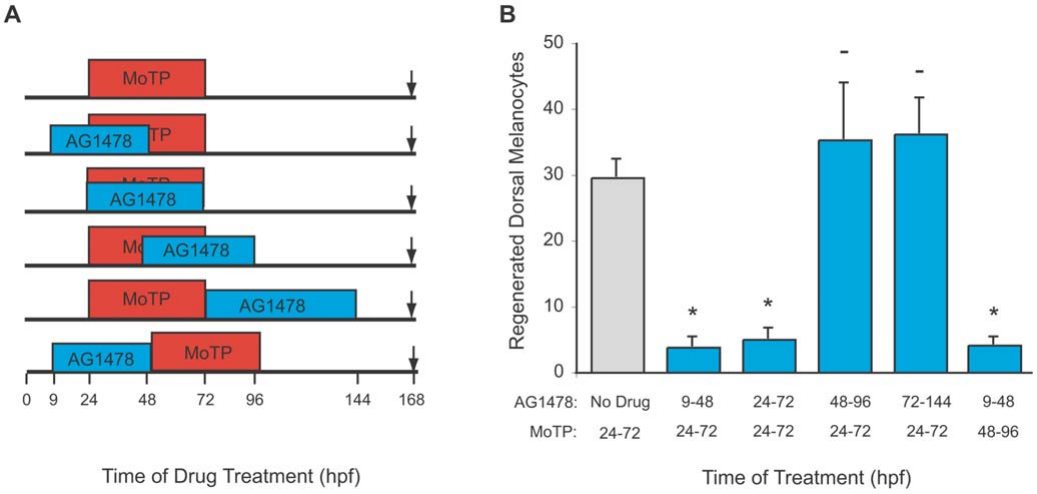
Regeneration requires ErbB signaling before 48 hpf. (A) Cartoon of drug treatment timeline. (B) Quantitation of average regenerated dorsal trunk melanocytes from somites 5–12 for each treatment in (A) for untreated (gray) and AG1478 treated (blue). Error bars represent standard deviation, * P<0.05, - P>0.05 (Student t-test, N = 10). Time shifts with AG1478 treatment reveal that it blocks regeneration when administered prior to 48 hpf. When AG1478 is added after 48 hpf regeneration is unaffected. The early effect is observed when MoTP treatment is withheld until after AG1478 washout at 48 hpf.

The early requirement for ErbB activity in promoting metamorphosis suggested that it was required to establish a population of precursors for adult melanocytes. To determine whether ErbB signaling has a similarly early role in establishing stem cells important for larval regeneration, we treated fish with AG1478 at various time intervals both before and after MoTP treatment and examined the number of dorsal trunk melanocytes (somites 5–12) that regenerated (Figure 2A). We found that incubations with AG1478 from 9–48 hpf and 24–72 hpf resulted in reduced numbers of regenerated melanocytes (3.8±1.8 and 5.0±2.1 dorsal trunk melanocytes, respectively, Figure 2B) compared to untreated controls (29.6±4.4 dorsal trunk melanocytes). In contrast, treatment with AG1478 after 48 hours resulted in no significant effect on regeneration (35.3±13.8 dorsal trunk melanocytes, Figure 2B). These results suggest that AG1478 acts prior to 48 hours to block melanocyte regeneration after the MoTP-induced melanocyte ablation.

In the foregoing analysis, the ErbB inhibitor AG1478 was applied in a series of applications overlapping with the embryo's exposure to the melanocyte ablation agent, MoTP. In those experiments, melanocytes or melanoblasts likely begin to die early in the MoTP treatment, between 24 and 48 hpf [27], thus relieving their repressive effect on melanocyte stem cell recruitment during the AG1478 treatment. It therefore remained possible that the effect of AG1478 in these experiments was to block recruitment of the MSC into developmental pathways following MoTP-induced ablation, rather than preventing the establishment of the MSC. To rule out this possibility, we delayed application of MoTP until 48 hours, after AG1478 was washed out. Like earlier MoTP exposure, melanoblasts and melanocytes are ablated by exposure to MoTP between 48 and 96 hours. Following washout of the MoTP, few new melanocytes regenerated (4.0±1.5 dorsal trunk melanocytes, Figure 2B). This result argues that AG1478 is acting during stem cell establishment stage to deplete the melanocyte stem cells and prior to any relief of repression and subsequent recruitment that follows from the delayed ablation of embryonic melanoblasts and melanocytes.

Although the above experiment positively demonstrates the effect of AG1478 on blocking MSC establishment, it does not exclude an additional effect of AG1478 on preventing recruitment of the MSC following melanocyte ablation. To rule out such a role, we performed a reciprocal experiment, where MoTP was applied first, from 24–72 hpf, followed by AG1478 treatment from 72 to 144 hpf. In these larvae, melanocytes regenerated in identical numbers to control larvae that were only treated with MoTP (Figure 2B). This result indicates that AG1478 has no effect on the recruitment of the melanocyte stem cell to re-enter developmental pathways when the repressive effects of melanocytes and melanoblasts are relieved.

The preceding analyses, which show that AG1478 acts on the MSC before embryonic melanocytes and melanoblasts are ablated, argue that ErbB signaling acts to promote the establishment of the melanocyte stem cell population rather than on recruitment or differentiation of melanocytes from the stem cell population during larval melanocyte regeneration. This conclusion is consistent with previous studies that showed that AG1478 treatment during early embryonic stages was sufficient to block melanocyte development during pigment pattern metamorphosis nearly two weeks later [23]. Together, these studies provide conclusive evidence that AG1478 blocks establishment during embryonic stages of an adult stem cell, the MSC, that is responsible for larval melanocyte regeneration and pigment pattern metamorphosis.

### Stem Cell Establishment Occurs in Anterior-to-Posterior Progression

**Figure 3:**
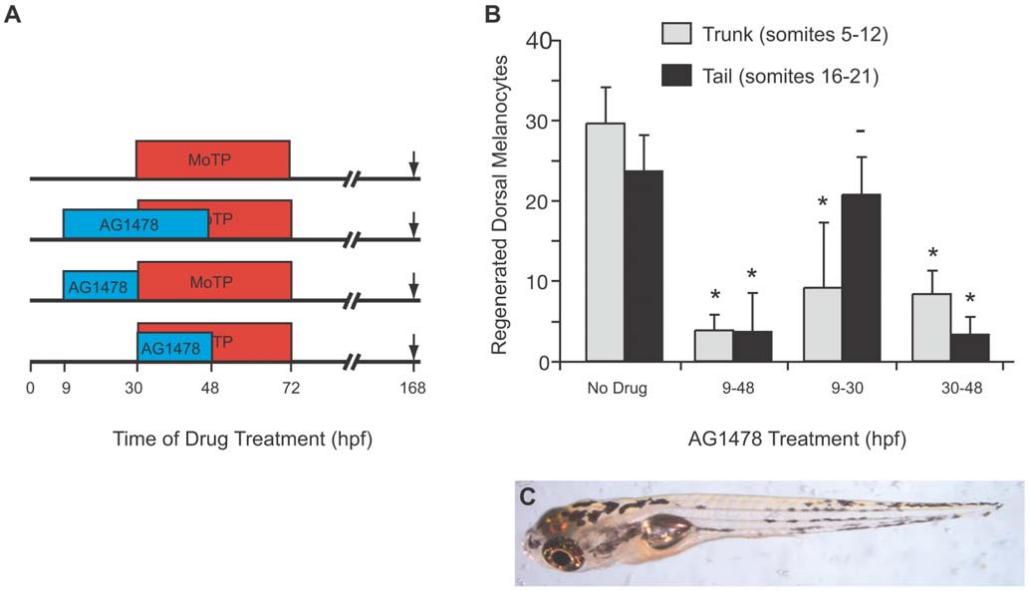
Temporal shifts of AG1478 reveal stem cell establishment occurs in a rostrocaudal progression. (A) Cartoon of drug treatment timeline. (B) Quantitation of average regenerated dorsal melanocytes for each treatment in (A) in the trunk (somites 5–12, in gray) and in the tail (somites 16–21, in black). Error bars represent standard deviation, * P<0.05, - P>0.05 (Student t-test, N = 10). Larvae not treated with AG1478 show full regeneration in the trunk and in the tail. When larvae are treated with the full AG1478 treatment, from 9–48 hpf, they fail to regenerate in either the trunk or the tail. Larvae treated early with AG1478 from 9–30 hpf fail to regenerate in the trunk, but have normal regeneration in the tail. Later treatments of AG1478 from 30–48 hpf show more regeneration in the trunk than in the tail. (C) Larva with early treatment of AG1478 from 14–24 hpf showing a regeneration defect in the trunk but with normal regeneration in the head and tail.

Because many aspects of zebrafish embryonic development and neural crest development proceed in rostrocaudal progression [7],[28],[29], we asked whether MSC establishment also proceeded similarly. Accordingly, we incubated fish in AG1478 for shorter times during the 9–48 hpf window that we showed was sufficient for complete or nearly complete inhibition of dorsal melanocyte regeneration (Figure 3A). Embryos exposed to AG1478 from 9–30 hpf showed nearly complete inhibition of regeneration in dorsal melanocytes in the trunk (9.1±8.0 melanocytes in somites 5–12), but no deficit in the tail (20.7±4.6 melanocytes in somites 16–21). In contrast, exposure to AG1478 from 30–48 hpf results in a profound deficit in tail melanocyte regeneration (3.3±2.1 melanocytes in somites 16–21). This result suggests that MSCs are established in a temporal rostral-caudal gradient similar to that observed in zebrafish neural crest development [7],[29].

### ErbB Signaling Is Not Required for Stem Cell Self-Renewal

**Figure 4:**
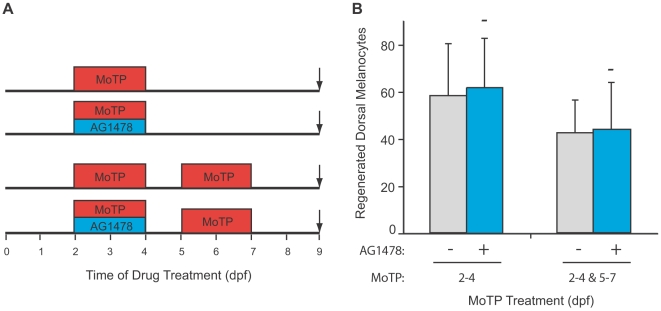
ErbB signaling is not required for stem cell self-renewal. (A) Cartoon of drug treatment timeline for single and double regeneration assays. (B) Quantitation of average regenerated dorsal melanocytes for each treatment in (A). Error bars represent standard deviation, - P>0.05 (Student's t-test, N = 10 for single regeneration, >5 for double regeneration). As previously shown (see Figure 2), when larvae are treated with AG1478 after 2 dpf, they regenerate normally following a single round of MoTP treatment. When animals are treated with two rounds of MoTP (from 2–4 dpf and from 5–7 dpf) they regenerate slightly less melanocytes than a single round by 9 dpf. To test whether AG1478 treatment would block stem cell self-renewal, we treated animals with MoTP and AG1478 from 2–4 dpf, and then with a second round of MoTP from 5–7 dpf. These animals regenerated melanocytes similar to non-AG1478 treated larvae for double regeneration (P = 0.9).

We were also interested in testing whether the ErbB inhibitor AG1478 blocked the mechanism of stem cell self-renewal that we postulate to occur during melanocyte regeneration. We have shown that following multiple rounds of MoTP-induced melanocyte ablation [27], melanocytes continue to regenerate or undergo pigment pattern metamorphosis. This property suggests that the melanocyte stem cell that supports larval melanocyte regeneration is not exhausted by making new melanocytes, but rather replaces itself, or self-renews, each time it is recruited to divide and make new melanocytes. We reasoned that these self-renewing divisions may have properties similar to the mechanisms that establish the stem cell during embryonic stages, and thus, might also require ErbB signaling. Such a requirement for ErbB signaling has been demonstrated for the in vitro self-renewal of human embryonic stem cells [30]. Moreover, we reasoned that post-establishment application of a drug that blocked self-renewal would allow for a first round of melanocyte regeneration, but show deficits in subsequent rounds of regeneration. Accordingly, we developed a double regeneration assay (Figure 4A), by which melanocytes are first simultaneously treated between 48 and 96 hpf with MoTP (to ablate melanoblasts and melanocytes) and AG1478, to potentially prevent self-renewal during this period. We had previously shown that MSC division, and thus self-renewal, begins within 24 hours of application of MoTP to the embryo [27], and also that AG1478 treatment at this stage has no effect on MSC recruitment or differentiation (see above). The melanocytes and melanoblasts that regenerate by 5 days are again ablated with a further 2 day exposure to MoTP, and again allowed to regenerate. In these experiments, we find that exposure to AG1478 during the first round of MoTP treatment has no effect on the second round of melanocyte regeneration (compare 44.0±19.8 dorsal melanocytes in AG1478-treated embryos to 42.6±13.8 dorsal melanocytes in MoTP-only-treated animals, P = 0.9, Figure 4B). Thus, these experiments suggest either that ErbB signaling is not required for MSC self renewal, or, less likely, that a different population or subset of MSC are deployed following each round of melanocyte ablation and regeneration.

### Overexpression of *kit ligand a (kitla)* Results in Overproliferation of Ontogenetic Melanocytes

We were interested in the role of the *kit* receptor tyrosine kinase in stem cell function. Previous work with conditional mutations in *kit* have shown that, although *kit* activity is required for regeneration, *kit* does not play an essential role in establishing MSCs, but is required for early stages in the development of melanocytes from stem cell precursors [13],[31]. Additionally, we previously reported that transiently overexpressing the *kit* ligand, *kitla* in embryos results in 50% more melanocytes than wild type [15]. We therefore wanted to investigate whether *kitla* overexpression might be recruiting the MSC to overproliferate or generate excess melanocytes.

**Figure 5:**
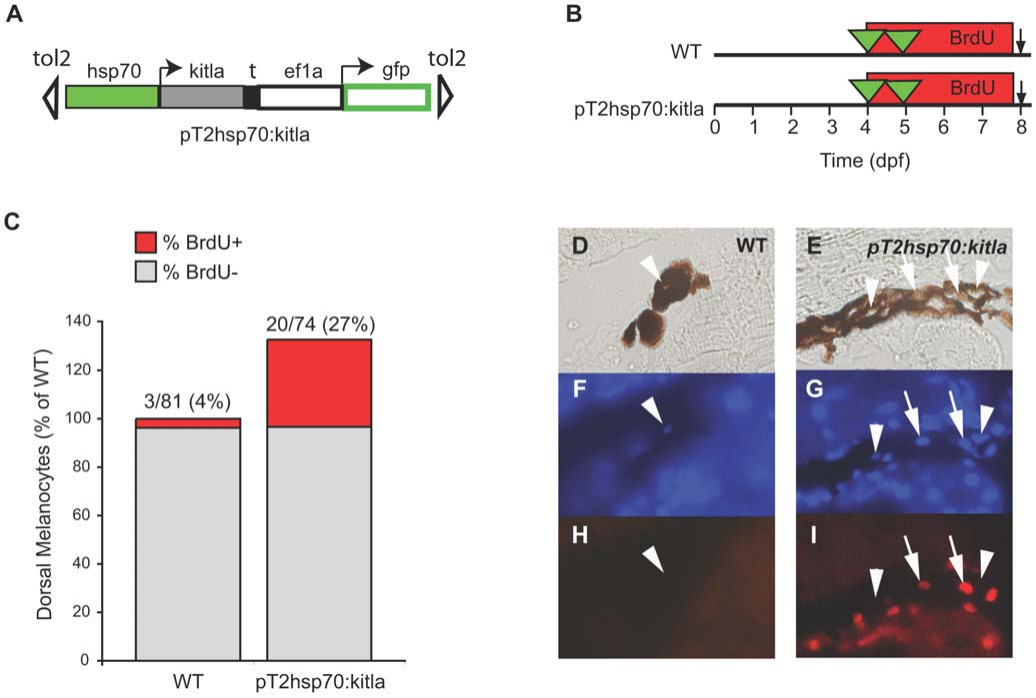
Overexpression of *kitla* after 4 dpf results in proliferation of melanocytes. (A) Cartoon of the *pT2hsp70:kitla* expression construct used in heatshock experiments. The heatshock promoter, hsp70 drives *kitla* expression, allowing for expression of *kitla* after heatshocking the injected embryos at 37°C. (B) Cartoon of experimental protocol. Following injection of *pT2hsp70:kitla*, larvae were heatshocked for 1 hour at 4 and 5 dpf in the presence of BrdU (from 4 to 8 dpf). (C) Quantitation of dorsal melanocytes represented as a percentage of wild type, and the percentage of BrdU labeled melanocytes is represented in red for each treatment. Larvae injected with *pT2hsp70:kitla* and heatshocked develop approximately 30% more melanocytes than uninjected larva. These larvae also show an increased percentage of BrdU labeled melanocytes (∼27%) that is comparable to the number of excess melanocytes. Representative examples of melanocytes in WT (D, F, H) and *pT2hsp70:kitla* (E, G, I), showing melanocyte nuclei stained with DAPI for WT (F) and *pT2hsp70:kitla* (G), and BrdU staining for WT (H) and *pT2hsp70:kitla* (I). Melanocytes considered having BrdU+ nuclei are labeled with arrows and BrdU- nuclei are labeled with arrowheads.

We asked whether *kitla* overexpression is acting on a transient population of melanoblasts during the ontogenetic development of melanocytes in the embryo. Wild-type animals develop nearly all of their embryonic melanocytes by 3 dpf. These differentiated melanocytes are post-mitotic, and there is little or no turnover of melanocytes between 3 dpf and the onset of metamorphosis at 14 dpf [12]. Therefore, to rule out that *kitla* overexpression is acting on a transient population of melanoblasts that are present only from neural crest appearance at 14 hpf to, at the latest, completion of embryonic melanocyte patterning at 72 hpf, we induced *kitla* expression using the heat shock promoter *(pT2hsp70:kitla)* in transiently injected embryos after 3 dpf. When injected embryos are heatshocked at 4 and 5 dpf, we observed an increase of 30% in the number of melanocytes by 8 dpf (227.9±18.3 dorsal melanocytes compared with 172.0±13.7 dorsal melanocytes in WT, P<0.05, Figure 5). Injected but non-heatshocked animals showed no significant increase in the number of melanocytes (176.8±18.3 dorsal melanocytes) compared with uninjected animals (P = 0.5, not shown). This result shows that the precursors that give rise to excess melanocytes remain available to *kitla* recruitment after embryonic melanocyte development is completed at 72 hpf.

We next sought to determine whether *kitla* overexpression caused an increase in melanocyte number by inducing cell proliferation. We have previously shown that regenerated melanocytes incorporate BrdU labeling after MoTP addition [13], indicating that MoTP-induced stem cell recruitment involves cell division. Whereas MSC recruitment is thought to involve a step in cell division, the excess cells following overexpression of *kitla* may arise by an alternative mechanism not involving cell division, such as the direct induction of latent melanoblast differentiation. Therefore, we again injected the heatshock *kitla* overexpression construct, *pT2hsp70:kitla*, into embryos and then treated the larvae with BrdU for 4 days beginning at 4 dpf (Figure 5). We then subjected *pT2hsp70:kitla*-injected and uninjected animals to heatshock treatment at 4 and 5 dpf and assayed for BrdU-positive melanocytes at 8 dpf (Figure 5A). In non-injected animals, 3 out of 81 (4%) melanocyte nuclei examined had BrdU staining. This low fraction of BrdU-labeled cells was expected as the vast majority of these melanocytes were born (and post-mitotic) before the BrdU incubation. In larvae injected with *pT2hsp70:kitla*, however, 20 out of 74 (27%) melanocyte nuclei examined had BrdU label. This fraction is nearly identical to the fraction of excess cells we observe for this heatshock treatment (∼30%), indicating that cell division accounts for all of the ectopic melanocytes observed in *pT2hsp70:kitla* heatshocked embryos (Figure 3B). We conclude that *kitla* overexpression causes an excess of melanocytes through a mechanism that requires cell division.

**Figure 6:**
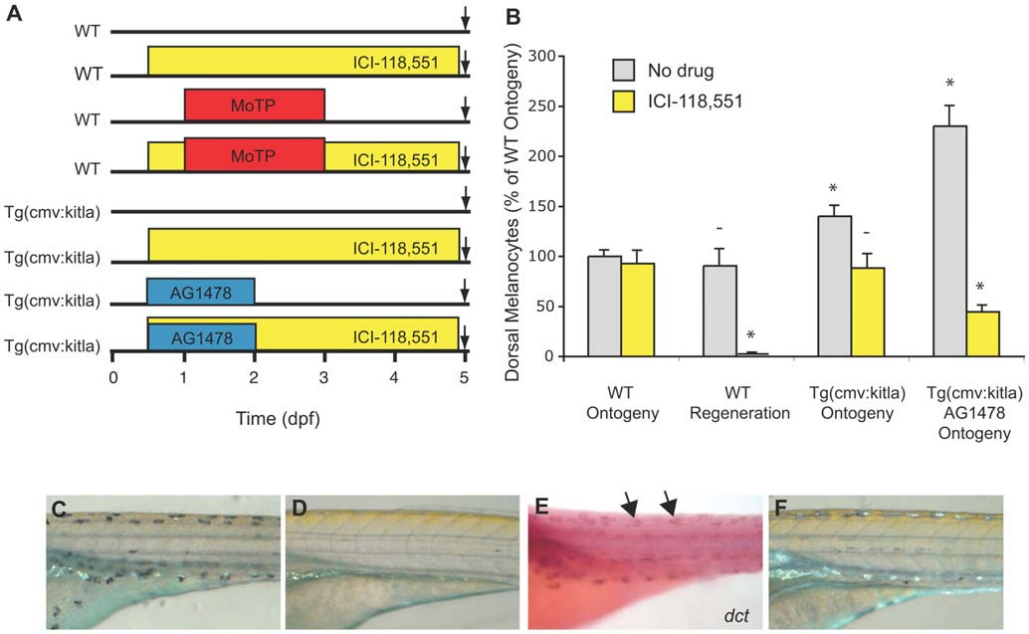
The regeneration specific drug, ICI-118,551, reveals that excess melanocytes in *PT2hsp70:kitla* larvae arise from the MSC lineage. (A) Cartoon of drug treatment timeline. (B) Quantitative data for ontogeny and regeneration represented as percentage of WT melanocyte numbers. Error bars show standard deviations, * P<0.05, - P>0.05 (Student's t-test, N>7). (C) WT larvae treated with ICI-118,551 develop faintly melanized ontogenetic melanocytes, in contrast to (D) failure to develop melanized melanocytes when challenged to regenerate in the presence of ICI-118,551. (E) *In situ* analysis reveals regeneration proceeds to the *dct*+ melanoblast stage (arrows) in the presence of ICI-118,551. (F) ICI-118,551 treated *PT2hsp70:kitla* embryos develop similar numbers of faintly melanized melanocytes as ICI-118,551 treated embryos shown in (C). Differences in iridophore appearance between (C) and (F) are results of slightly different illumination conditions.

**Figure 7:**
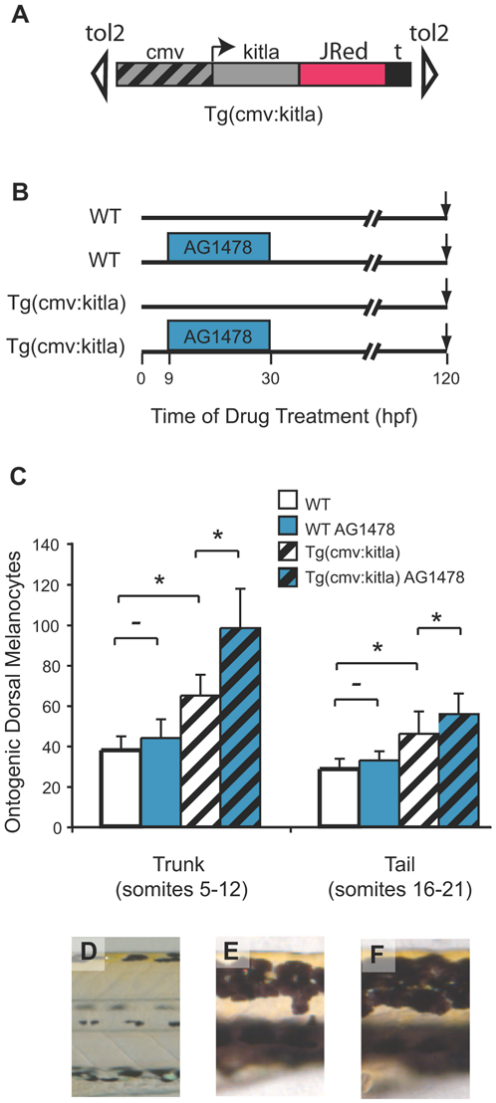
*kitla* melanocyte proliferation is enhanced by AG1478. (A) Cartoon of the *PT2hsp70:kitla* construct in transgenic animals. The constitutive cmv promoter drives expression of *kitla*. (B) Schematic of drug treatment timeline with early treatment of AG1478. (C) Quantitation of average ontogenetic dorsal melanocytes for each treatment in (A) in the trunk (somites 5–12) and in the tail (somites 16–21). Error bars represent standard deviation, * P<0.05, - P>0.05 (Student t-test, N = 10). As reported previously (see Figure 1) WT larvae show no effect in ontogenetic melanocyte number with AG1478 treatment compared to untreated WT. *PT2hsp70:kitla* animals have significantly more melanocytes in both the trunk and the tail. Treating *PT2hsp70:kitla* animals with AG1478 early, from 9–30 hpf, produces a significantly greater number of melanocytes in the trunk. In contrast, the tail region of AG1478-treated *PT2hsp70:kitla* larvae shows a significant, but much smaller increase than the trunk, in the number of excess melanocytes. (D) Trunk dorsal stripes of WT larvae are typically two melanocytes wide. (E) *PT2hsp70:kitla*, however, have trunk dorsal stripes that are about 4 cells wide. (F) AG1478-treated *PT2hsp70:kitla* have trunk dorsal stripes ∼6 melanocytes wide.

To rule out the possibility that the above demonstrated cell division events involve the cell division of differentiated melanocytes in response to overexpression of *kitla*, we asked whether addition of the tyrosine kinase inhibitor, phenylthiourea (PTU), after 3 dpf could inhibit the appearance of these excess melanocytes. For this experiment we used a stable transgenic line of fish that contain *kitla* under the control of the cmv promoter *(Tg(cmv:kitla)). Tg(cmv:kitla)* animals have ubiquitous *kitla* mRNA expression (not shown) and, similar to the previously reported transient experiments, have ∼50% more melanocytes than wild type (see Figure 6 and Figure 7). In the presence of PTU, melanocytes develop, but fail to form pigment. In these experiments, we observed no increase in the number of pigmented melanocytes in PTU-treated *Tg(cmv:kilta)* larva by 6 dpf (data not shown), indicating that *Tg(cmv:kitla)*-induced melanocyte increase is not a consequence of cell division of melanized melanocytes. We therefore conclude that *kitla* recruits undifferentiated, or nonpigmented, cells to divide and differentiate into melanocytes. That these cells can be recruited by overexpression of *kitla* at 4 or 5 dpf, when *in situ* expression analysis reveals no *dct+*, melanin- melanoblasts present, suggests that excess *kitla* is acting on an earlier stage of melanocyte development. A candidate for a cell capable of recruitment by *kitla* is the MSC.

### ICI-118551 Prevents Differentiation of Melanocytes During Regeneration, But Not Ontogeny

To establish that ectopic overexpression of *kitla* increases the production of melanocytes through recruitment of the MSC lineage, rather than the embryonic melanocyte lineage, we asked whether this increase was sensitive to the MSC lineage-specific drug, ICI-118,551. We identified ICI-118,551 in a screen for small molecules that specifically blocked melanocyte regeneration following MoTP induced ablation (A. Gottlieb and S. Johnson, unpublished data). Ontogenetically developing embryonic melanocytes develop normally, albeit with fainter pigment, in the presence of ICI-118,551 (Figure 6C). In contrast, larvae challenged to regenerate their melanocytes in the presence of ICI-118,551 develop *dct*+ positive melanoblasts (Figure 6E), but these melanocytes remain unmelanized (Figure 6D). This difference in effect of ICI-118,551 in ontogenetic and regeneration melanocytes is similar to the effect of the *gfpt1 (eartha)* mutation, which also blocks melanocyte regeneration at the *dct+* melanoblast stage and has lighter pigmented ontogenetic melanocytes than WT [14]. Thus, sensitivity of melanocytes to ICI-118,551 can be used to distinguish whether melanocytes arise from the primary embryonic melanocyte lineage or from the MSC lineage. ICI-118,551 is a β2-adrenergic receptor antagonist in mammals and fish, acting to block β2-adrenergic receptor-mediated cAMP increase in fish larvae. This is easily observed by its effects on contracting the melanosomes in the melanocyte. It is unlikely that ICI-118,551's effects on regeneration are mediated through β2-adrenergic receptors, as other β2-adrenergic receptor antagonists, which are effective in contracting melanosomes in fish, have no effect on regeneration (not shown). Additionally, treating embryos together with ICI-118,551 and Forskolin, which should result in elevated cAMP levels and blocks the effect of ICI-118,551 on melanocyte contraction, fails to block the effect of ICI-118,551 on melanocyte regeneration. Thus, ICI-118,551 may be acting on a yet identified pathway to specifically block zebrafish melanocyte development from the melanocyte stem cell.

### ICI-118,551 Suppresses *kitla*-Induced Overproliferation of Melanocytes

To test whether *kitla* overexpression is recruiting melanocytes from the stem cell lineage rather than from the ontogenetic melanocyte lineage, we treated *Tg(cmv:kitla)* animals with ICI-118,551 beginning at 9 hpf (Figure 6A) and asked whether the drug could block differentiation of excess melanocytes. Indeed, the number of melanocytes in ICI-118,551 treated *Tg(cmv:kitla)* larva (169.1±26.3 dorsal melanocytes, Figure 6B and 6F) is less, but not significantly different, from that of WT (191.4±11.6, P = 0.06). This result indicates that the excess melanocytes in *Tg(cmv:kitla)* animals develop from the stem cell lineage rather than from the ontogenetic lineage.

### AG1478 Enhances the Ectopic Melanocyte Phenotype of *kitla* Overexpression

We were next interested in how AG1478 is acting to inhibit MSC establishment. Specifically, we wanted to know whether AG1478 killed the stem cell lineage, or whether it blocked the MSC from developing from a pre-stem cell (pre-MSC) state. We suspected that AG1478 prevented cells in the neural crest lineage from achieving melanocyte stem cell specification, and that if these precursors were still alive, they might be expressing *kit* [18], and therefore might still be capable of recruitment through overexpression of *kitla*. To test whether *kitla* could induce AG1478-treated larvae to produce excess melanocytes, we treated wild-type and *Tg(cmv:kitla)* transgenic larvae with AG1478 from 9–30 hpf (Figure 7B). This time period of AG1478 treatment prevented melanocyte regeneration in the trunk but not the tail of larvae (see Figure 3), providing an internal comparison between regions with affected and less affected stem cells in the larvae. As noted earlier, wild type larvae treated with AG1478 show no difference in the number of melanocytes that develop in either the trunk or the tail (Figure 7C). Untreated *Tg(cmv:kitla)* larva show an increase in the number of melanocytes in both the trunk (64.8±9.4 dorsal melanocytes, or 171% of wild type, Figure 7C) and the tail (47.9±12.2 dorsal melanocytes, or 168% of wild type, Figure 7C). When *Tg(cmv:kitla)* larva are treated with AG1478, they show an additional increase of 52% in the number of melanocytes in the trunk (98.2±18.6 dorsal melanocytes, Figure 7C) compared with untreated *Tg(cmv:kitla)* (P<0.0001). In contrast to the two-melanocyte width of the dorsal stripe typically observed in wt larvae (Figure 7D), dorsal melanocyte stripes in *Tg(cmv:kitla)* (Figure 7E), or AG1478 treated, *Tg(cmv:kitla)* (Figure 7F) larvae are often four or six melanocytes wide, respectively. In these experiments, this effect is much less pronounced in the tail of AG1478-treated *Tg(cmv:kitla)* larvae (55.6±9.3 melanocytes, Figure 7C). Compared with untreated *Tg(cmv:kitla)* (45.2±6.7 melanocytes, Figure 7C) this effect is less than a 20% increase (P<0.05). That AG1478 enhances *Tg(cmv:kitla)*-induced overproduction of melanocytes primarily in the trunk region where AG1478 prevents the establishment of MSCs reinforces our conclusions developed above that *kitla* overexpression is acting on the MSC lineage, rather than embryonic melanoblasts and melanocytes, to cause overproduction of melanocytes.

To rule out the possibility that *kitla* is recruiting cells from the ontogenetic lineage when the MSCs are blocked with AG1478, we treated *Tg(cmv:kitla)* larvae with both AG1478 and ICI-118,551 (See Figure 6A and 6B). When *Tg(cmv:kitla)* larva are treated with AG1478 and ICI-118,551 together, we observed even fewer ontogenetic melanocytes (44.6% of that of WT). This result provides further evidence that *kitla* is capable of recruiting the AG1478 pre-MSC. That fewer ICI-118,551-insensitive melanocytes develop in *Tg(cmv:kitla)* AG1478-treated larva might suggest that the proliferation of the ontogenetic melanoblast lineage is suppressed when the stem cell lineage is overproliferating to such an extent (230% of wild type).

Taken together, four lines of evidence converge to indicate that the excess melanocytes that develop following overexpression of *kitla* arise from the MSC lineage, rather than the ontogenetic (ErbB-independent) lineage. These include (1) overexpression of *kitla* is capable of inducing an excess of melanocytes after 4 dpf when embryonic melanoblasts are not present; (2) all excess melanocytes arise through cell division after *kitla* overexpression; (3) ICI-118,551, which does not affect embryonic melanocyte number, blocks the increase in *kitla* overexpressing animals and lastly; (4) AG1478, acts specifically in the MSC lineage, modifies the number of excess melanocytes in *kitla* overexpressing animals.

The foregoing analysis now allows us to draw two conclusions. First, it suggests that AG1478 treatment does not kill the MSC, but rather results in a cell that is now recruitable by excess *kitla*. This cell does not have the full regulative abilities of a MSC, as evidenced by the fact that it cannot respond to ablation of embryonic melanocytes by making new melanocytes. Its presence, rather than immediate death, is revealed by its responsiveness to excess *kitla*. A parsimonious interpretation is that AG1478 prevents—and *erbb3b* is required for—the progression of a precursor to the fully regulative MSC. The second conclusion is that this precursor, that we refer to as a pre-MSC, is responsive to *kit* signaling. Together with the findings that *kitla* overexpression after 4 days induces overproduction of new melanocytes through cell division of undifferentiated precursors (see Figure 5) from the MSC lineage (see Figure 6) now provides functional evidence for a role of *kit* signaling in both the pre-MSC and the MSC itself in recruiting these cells to divide and produce more melanocytes.

## Discussion

**Figure 8:**
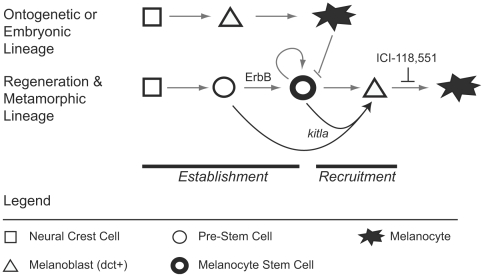
Model for the parallel establishment of the zebrafish embryonic melanocyte lineage and the adult melanocyte stem cell lineage. Our results indicate that there are two distinct melanocyte lineages that develop in the zebrafish embryo: the embryonic or ontogenetic melanocyte lineage and the regenerative and metamorphic melanocyte lineage. In embryonic development, neural crest cells (squares) give rise to *dct+* melanoblasts (triangles), which later develop into embryonic melanocytes (black star). The regeneration and metamorphic lineage develops in parallel to embryonic melanocytes, prior to 48 hpf and presumably from the neural crest. The establishment of melanocyte stem cells (MSCs, dark circle) require ErbB signaling to progress from the pre-MSC state (light circle). The MSC can self-renew (circular arrow) and give rise to melanoblasts and melanocytes during metamorphosis or regeneration, when inhibition from embryonic melanocytes (block arrow) is relieved. The MSC–derived melanocytes are sensitive to developmental blockade by ICI-118,551 after the *dct*+ melanoblast stage. Ectopic expression of *kitla* can recruit the MSC lineage by either inducing MSCs or pre–MSCs resulting in overproliferation of melanocytes.

In this study, we have presented evidence for several conclusions regarding the origin and recruitment of zebrafish adult melanocyte stem cells (MSC). Our model for MSC establishment and recruitment is presented in Figure 8, and has four salient features. First, the establishment of the MSC requires ErbB activity to progress from a pre-MSC to a MSC state. Second, the MSCs responsible for regeneration in the larva may be identical to stem cells previously shown to be involved in metamorphic patterning of the adult fish [23]. Third, primary ontogenetic, or embryonic, melanocytes develop independently of the MSC. Fourth, ectopic overexpression of *kitla* can recruit both the MSC and the ErbB inhibitor-arrested pre-MSC. The broader implications for these findings are discussed in more depth below.

### Role of ErbB Signaling in the Origin of the Adult Melanocyte Stem Cell

We have both genetic and pharmacological evidence that ErbB signaling is required for the establishment of the adult melanocyte stem cell. Although we lack direct markers for the melanocyte stem cell, we can draw strong inferences about its existence and regulation from the ability of larvae to regenerate melanocytes, the formation of new melanocytes upon pigment pattern metamorphosis, or the regeneration of melanocyte stripes in the regenerating adult fin. We have previously shown that following ablation of embryonic melanocytes, cells with stem cell properties are recruited to replace or regenerate the missing melanocytes [13]. This notion is supported by BrdU incorporation experiments that showed that this cell and its daughters divide several times in response to embryonic melanocyte ablation [13]. Here we show that treatment with ErbB inhibitor prevents melanocyte regeneration. We have mapped the timing of ErbB inhibitor action and shown that ErbB signaling is required during periods of embryonic development coincident with ontogenetic melanocyte migration and differentiation (14–48 hpf). That ErbB inhibitors are blocking the establishment of the MSC, rather than subsequent stages of MSC recruitment, renewal, or differentiation, is demonstrated positively by the fact that exposure of the larvae to the ErbB inhibitor prior to MoTP-induced melanocyte ablation blocks regeneration (Figure 2). That exposure to ErbB inhibitor during or after MoTP treatment has no effect on subsequent melanocyte regeneration suggests that ErbB signaling has no role in recruitment, renewal, or differentiation of this lineage. Thus, we conclude that the MSC is established during embryonic development during stages of melanocyte migration from the neural crest, and that establishment of the MSC requires ErbB signaling.

### Regeneration and Metamorphosis Depend on a Shared Melanocyte Stem Cell

Our finding that larval melanocyte regeneration was sensitive to ErbB signal inhibition led us to explore whether the *erbb3b* mutant was also defective for larval melanocyte regeneration following ablation with MoTP. We find that embryos treated with ErbB inhibitor and the *erbb3b* (*picasso*) mutant, which are both defective for metamorphic melanocyte development [23], are also defective for larval melanocyte regeneration. We also find that the ErbB inhibitor acted to block regeneration early, from 9–48 hpf, which is at a similar embryonic stage that it was found to prevent melanocyte development during metamorphosis (12–22 hpf). Together, these results suggest that both metamorphic melanocytes and regeneration melanocytes rely on the same ErbB-dependent MSCs. The alternative possibility is that the zebrafish has two distinct populations of MSCs established at similar times with similar requirements for ErbB signaling. The development of persistent labeling techniques for long-term clonal analysis will be required to investigate the relationship between different melanocyte populations to more firmly rule out this latter possibility.

Our interpretation that larval regeneration melanocytes and metamorphic melanocytes arise from the same adult stem cells sheds some light on how adult stem cells may be regulated by different mechanisms of repression or induction at different stages of development. During larval stages (3–14 days), melanocyte development is nearly static, with few or no new melanocytes developing during this period (but see [11]). That ablation of embryonic melanocytes during this stage activates the MSC argues that the MSC is repressed by differentiated embryonic melanocytes. Such repression might be direct interaction (as we propose in Figure 8) or mediated by other cells, such as a yet unidentified MSC niche, that in turn activates the MSC when the melanocyte is ablated. In contrast, metamorphosis is a concerted set of developmental events initiated by thyroid hormone [32] in the presence of embryonic melanocytes, some of which can persist into the adult pattern [21]. This argues that either the signals of metamorphosis override embryonic melanocyte repression of the MSC, or that embryonic melanocytes no longer repress the MSC during this developmental change. Thus, the differences in mechanisms between MSC recruitment during regeneration and metamorphosis will provide useful insights into the regulation of adult stem cells.

### Independent Lineage for Ontogenetic and Regenerative Melanocytes

This study now provides strong evidence for two distinct melanocyte lineages that populate the larval zebrafish. One melanocyte population, which we have discussed extensively in this study, is derived from an ErbB-dependent melanocyte stem cell (bold circle in Figure 8), and develops in response to ablation of earlier developing melanocytes. This earlier population, which we refer to as embryonic or ontogenetic melanocytes, develops directly from neural crest cells, and we argue does not develop from an adult stem cell-like intermediate. It is difficult to formally rule out such a stem cell precursor for the ontogenetic lineage, but it is clear from the fact that their development is unperturbed in mutants for *erbb3b* or following treatment with ErbB inhibitors that the ErbB-dependent MSC is not a precursor for ontogenetic melanocytes. Moreover, that the ErbB independent melanocyte precursors in the ontogenetic lineage cannot regulate the larval pigment pattern when the ErbB-dependent MSCs are blocked suggests that a precursor for these cells do not persist in the larva through self-renewal. Self-renewal, in contrast to depletion during development, is a hallmark for embryonic or adult stem cells [33].

The two distinct mechanisms for development, direct and stem cell-derived, have also been argued for the development of melanocytes in the mouse. In this case, the differentiated melanocytes of the first hair follicle are the result of direct development, while the melanocytes of subsequent hair follicle cycle are thought to derive from a MSC [18]. Thus, although the biology of embryonic pigmentation and metamorphosis in zebrafish superficially appears distinct from that of mammals, these two results now suggest that the embryonic melanocytes in zebrafish are analogous to those of the first hair follicle in mouse, and post-embryonic melanocytes that appear during regeneration or metamorphosis are analogous to the melanocytes of second and subsequent hair follicles. Further investigation of each model organism will determine the extent of the common requirements and potential homologous mechanisms and will allow us to draw stronger conclusions for understanding the origin of the MSC.

That both mammals and zebrafish appear to develop melanocytes directly during embryogenesis and then subsequently through adult stem cells raises the question of whether such modes of development are a general theme in metazoan development. Certainly in insects such as *Drosophila*, the embryo sets aside cells that will develop via imaginal discs to then replace the larval structure with the adult structures. The imaginal discs are not themselves stem cells, and their development in the adult is largely terminal. Yet in recent years, adult stem cells that provide for cell turnover in the gut and other tissues have been described, raising the possibility that the Drosophila embryo may also establish adult stem cells [34]–[36]. Vertebrates seem to lack anything like the imaginal discs of the fruit fly, and it may be that most post-embryonic growth comes from adult stem cells. A parallel example to what we describe here for embryonic (direct developing) and stem cell-derived melanocytes may be provided by the development of the blood. While the blood cells of the adult vertebrate, referred to as definitive hematopoiesis, develop from a well characterized adult stem cell called the hematopoietic stem cell (HSC), primitive hematopoiesis, because of its transient nature, has had no such self-renewing adult stem cell ascribed to it [37]. Clonal analysis in *Xenopus* with these two populations of blood origin indicates that the primitive and definitive blood are derived from independent lineages [38]. Similar clonal analysis will be required to further examine the relationship between the melanocyte stem cell we have described here and the embryonic melanocyte lineage. Exploration of other types of adult stem cells and their corresponding embryonic tissues will reveal whether parallel development of the embryo and the establishment of the adult stem cell from distinct lineages is a general theme in vertebrate development.

## Materials and Methods

### Fish Rearing and Stocks

Adult fish were maintained at 25–27°C, 14L∶10D standard treatment (Westerfield, http://zfin.org/zf_info/zfbook/zfbk.html). Embryos and larval fish were reared at 28°C in 2 ppt marine salt (Coral Life, Carson, CA) in carbon-filtered tap water. Larvae were fed rotifers after 5 dpf. Wild type embryos photographed were sjA (http://zfin.org). All quantitative analysis for wild-type fish was conducted with fish mutant for melanophilin (*mlpha*), which contracts melanosomes within the melanocyte but does not effect the number or pattern of melanocytes [39]. The *erbb3b* (*picasso*) mutant, which has normal number of embryonic melanocytes but fails to develop normal adult melanocyte patterning, was previously described [23].

### Pharmacological Inhibitors

The kinase inhibitors AG1478 (Calbiochem) and PD158780 (Calbiochem) were dissolved in dimethyl sulphoxide (DMSO) to make a stock solution (6 mM AG1478 and 600 µM PD158780) and stored at −20°C in a light protected vessel. Dose response testing (1, 3, 5, and 10 µM) revealed a strong effect with limited lethality at 3 µM for both drugs. Unless otherwise noted, embryos were incubated in Petri dishes with 3 µM AG1478 with 0.5% DMSO in 2 ppt marine salt water, with one embryo per ml. Embryos were kept in the dark during incubation to protect drugs from light degradation.

The regeneration blocker ICI-118,551 was identified in a small molecule screen for drugs that specifically inhibit larval regeneration. Embryos were placed in MoTP (50 µM) and each of the 1250 drugs (10 µM) in the LOPAC (Library of Pharmacologically Active Compounds, Sigma, St. Louis, MO) panel at 12 hpf in 96 well plates. MoTP was washed out of each well at 3 dpf and replaced with additional drug. Drugs that inhibited regeneration by 6 dpf were tested for their effects on ontogenetic melanocyte development. Embryos treated with 30 µM ICI-118,551, had a complete absence of regenerated melanocytes but displayed a normal number of ontogenetic melanocytes.

### MoTP-Induced Melanocyte Ablation

MoTP (4-(4-morpholinobutylthio)phenol) stock solution (50 mM) was made up in DMSO, and stored at room temperature in a light protected vessel. Embryos were incubated in 50 µM MoTP in 2 ppt water for 48 hour incubation periods and assayed for regeneration after at least an additional 48 hours post washout. Embryos were visually inspected for complete melanocyte death 24 hours after washout.

### Melanocyte Counts

To assay for either melanocyte ontogeny or regeneration quantitatively, we counted dorsal melanocytes. Unless otherwise noted, these include melanocytes along the dorsum of the larva from somites 1–26. In each Figure we present the mean number of dorsal melanocytes with error bars indicating standard deviation. P-values for statistical significance were obtained by using Student's t-test in Microsoft Excel.

### *kitla* Overexpression

Fish transgenic for an overexpression line of the *kit ligand a (kitla)* driven under the *cmv* promoter, *Tg(cmv:kitla)^j901^*, were created. The *cmv:kitla* construct previously described [15] was cloned into the tol2 transposable element using the pT2KXIG vector [40] at the XhoI and BglI sites *(pT2cmv:kitla)*. Capped transposase RNA was synthesized from pCS-TP, provided by K. Kawakami, using the mMessage mMachine SP6 RNA synthesis kit (Ambion, Austin TX). A transposase injection solution was prepared fresh each day of injecting composed of 25 mg/ml *pT2cmv:kitla* DNA, 25 mg/ml transposase mRNA, and 0.1% phenol red (Sigma, St Louis MO) in 1× Danieau buffer. Animals were injected with 1–2 nl of *pT2cmv:kitla* transposase injection solution at the 1 or 2 cell stage. The mosaic F_0_ animals were scored for *kitla* induced hyperpigmentation at 5 dpf and bred to sjA to test for germ line transmission. A single F_1_ male was selected that had robust hyperpigmentation phenotype in 50% of its offspring when crossed with sjA. Expression of *kitla* was examined with RNA *in situ* hybridization in these siblings. All embryos with *kitla* phenotype stained positive for ubiquitous *kitla* mRNA expression, whereas only ∼5% of animals scored as WT stained for ubiquitous *kitla* mRNA. Experiments presented here are with *Tg(cmv:kitla)^j901^*/+ F_4_ embryos.

For heat inducible *kitla* overexpression, *kitla* was placed under the control of the heatshock promoter, hsp70 [41], and cloned into the tol2 vector, pT2ef1:GFP. This construct has a lineage tracing GFP marker under constitutive expression by the *Xenopus* elongation factor 1 alpha promoter. Animals were injected with between 1–2 nl of 25 µg/ml *pT2hsp70:kitla* and 25 µg/ml transposase RNA at the 1 or 2 cell stage. Genomic integration of the *pT2hsp70:kitla* construct was determined by GFP expression at 24 hpf, typically observed in >95% of injected animals. Only GFP+ embryos were used in heatshock experiments. Heatshock activation of *kitla* expression was performed by placing embryos in 50 ml conical tubes with 25 ml embryo medium and placing the tubes in a 37°C water bath for 30 minutes. All of the *pT2hsp70:kitla* construct experiments presented here are using F_0_ mosaic embryos.

### BrdU Labeling

Cell division events in the melanocyte lineage were detected by retention of 5-bromo-2′-deoxyuridine (BrdU). Animals mosaic for *pT2hsp70:kitla* genomic integration were immersed in BrdU (5 mM) for 96 hours beginning at 4 dpf. To induce *kitla* expression, animals were heatshocked at 4 and 5 dpf. Animals were then harvested at 8 dpf and fixed with 4% paraformaldehyde in phosphate buffered saline buffer (pH 7). More than 20 animals for each treatment were embedded in paraffin wax and sectioned into 5-micron-thick sagittal sections. Sections were deparaffinized and incubated with mouse anti-BrdU monoclonal antibody (1∶300 dilution, Roche Diagnostics, Indianapolis, IN), followed by a secondary incubation with Alexa Fluor 594-conjugated goat anti-mouse Ig (1∶300 dilution, Invitrogen), and 4′,6-diamidino-2-phenylindole (DAPI, 100 ng/ml; Sigma, St. Louis, MO) for nuclear staining [13]. BrdU incorporation was determined by first identifying DAPI-positive nuclei (blue fluorescence) in pigmented melanocytes, then assessing BrdU incorporation (red fluorescence) in each identified nucleus. Example nuclei that were categorized as BrdU negative (arrowheads) and BrdU positive (arrows) are shown in Figure 5C–5H.

### RNA *In Situ* Hybridization

RNA *in situ* analysis was used to asses the presence of *dct*-expressing melanoblasts. Larvae were fixed in 4% paraformaldehyde in phosphate buffered saline (pH 7) at 5 dpf. cDNA for *dct* was amplified using PCR primers 5′-TACCTGTCGGAGGCTCAGTT-3′ and (with T7 sequence in lowercase) 5′-taatacgactcactatagggTACGCTGGTCACAGTTCAGC-3′ from first strand cDNA of 48 hpf embryos. Digoxygenin (DIG) labeled RNA probes from *dct* cDNA template were made using T7 RNA polymerase using the protocol at http://zfish.wustl.edu. Anti-DIG *in situ* hybridization was performed as previously described [42].
